# Gender‐based violence and the role of healthcare professionals

**DOI:** 10.1002/nop2.120

**Published:** 2017-12-21

**Authors:** Parveen Ali

**Affiliations:** ^1^ Nursing and Midwifery University of Sheffield Sheffield UK

Academics, researchers, practitioners and millions of people from around the world participated in the violence against women campaign that ran from 25 November—International Day for the Elimination of Violence against Women—to 10 December—Human Rights Day. The campaign called “16 Days of Activism against Gender‐Based Violence” is an annual event that connects these two important dates and reminds the world that violence against women is a human rights issue. Therefore, this is an opportunity to write on the issue of gender‐based violence and its various forms to draw the attention of nurses, midwives and other healthcare professionals’ and the readers of *Nursing Open* to this subject.

The United Nations (1993) defines violence against women as “any act of gender‐based violence that results in, or is likely to result in, physical, sexual or mental harm or suffering to women, including threats of such acts, coercion or arbitrary deprivation of liberty, whether occurring in public or in private life” (United Nations, 1993). More recently, in the UK, the definition was revised to include controlling and coercive control referring to: “a range of acts designed to make a person subordinate and/or dependent by isolating them from sources of support, exploiting their resources and capacities for personal gain, depriving them of the means needed for independence, resistance and escape and regulating their everyday behaviour” (Home Office, 2012). Therefore, any “act or a pattern of acts of assault, threats, humiliation and intimidation or other abuse that is used to harm, punish, or frighten their victim” is an example of coercive control, and a punishable offence in the UK (Home Office, 2012).

Of course, men can also be victims of violence, although available evidence suggest that frequency, severity and intensity of such violence is much greater for women than men. Also, in most cases, the perpetrator of abuse is usually someone known to the woman with current or former intimate partner and other family members likely to be the most common perpetrator. Women are subjected to various acts of violence in all aspects of life. It happens in public as well as private, at home, in the street, in the office, in peace and in war. It takes many forms, including physical, psychological and sexual abuse. It affects girls and women of all ages, in the form of female infanticide, female genital mutilation, child marriage, grooming, trafficking, forced marriage, honour killing, domestic violence and intimate partner violence. It is associated with significant physical, emotional and mental health impacts. It not only has an impact on the lives of women experiencing it, but also negatively affects their children.

Disclosing abusive experiences to health and social care practitioners or police is not straightforward for women as it can create a sense of shame, embarrassment, powerlessness and hopelessness (Briones‐Vozmediano, Agudelo‐Suarez, Goicolea, & Vives‐Cases, 2014; Ortiz‐Barreda et al., 2014). However, there are subgroups of women who may experience additional difficulties when it comes to seeking support and these include older women (McGarry, Ali, & Hinchliff, 2017; McGarry & Bowden, 2017; Straka & Montminy, 2006), women from minority ethnic background (Ortiz‐Barreda et al., 2014; Stockman, Hayashi, & Campbell, 2015), women with disabilities (Dixon & Robb, 2015; Khalifeh et al., 2015) and those in institutional setting.

In recent years, a lot has been done to recognize and criminalize the issue by developing appropriate laws against it, although implementation of these laws is still challenging in many parts of the world. At the same time, a lot has been done to increase awareness of the general public as well as healthcare professionals about the issue in an attempt to prepare practitioners to identify and respond appropriately to those who need help. However, much still needs to be done.

We know that healthcare professionals including nurses and midwives work in diverse settings and can contribute by recognizing the manifestations and referring victims to appropriate sources of help and support (Ahmad, Ali, Rehman, Talpur, & Dhingrra, 2016). They need to be able to provide empathetic and supportive care to those who may be experiencing abuse to enable them to seek help and support. Nurses and other healthcare professionals need appropriate knowledge and skills to enable this. Active listening, an empathetic and non‐judgemental attitude and an awareness of one's own values and beliefs related to violence against women, prejudice and biases is necessary. Those nurses working in leadership positions can play their part by contributing to the development and implementation of appropriate policies, guidelines and legislations. Finally, nurses’ work and role exposes them to additional pressures as they not only care for those experiencing abuse, but at times, may have to care for those who perpetrate abuse on others and in such situations having self‐awareness, emotional intelligence and a knowledge of professional codes of conduct is useful. Identifying and helping perpetrators who recognize their behaviour and would like support to change their behaviour is also essential and nurses and healthcare professionals can do that by referring them to appropriate support programmes. Violence against women is a complex and multifactorial issue needing a multisectoral and ecological approach to deal with it and we as healthcare professionals can play a very important role in preventing it.
